# Precision in Practice: A Systematic Review and Meta-Analysis of Intraoperative Neurophysiological Monitoring for Optimizing Outcomes in Extramedullary Spinal Cord Tumor Resection

**DOI:** 10.3390/jpm15110513

**Published:** 2025-10-30

**Authors:** Raja Narendra Divakar Addanki, Benjamin B. Lee, Katherine M. Anetakis, Jeffrey R. Balzer, Parthasarathy D. Thirumala

**Affiliations:** 1 Department of Neurosurgery, University of Pittsburgh Medical Center, Pittsburgh, PA 15213, USA; addankirn@upmc.edu (R.N.D.A.); leebb@upmc.edu (B.B.L.); melonakoskc@upmc.edu (K.M.A.); balzerjr@upmc.edu (J.R.B.); 2 Department of Neurology, University of Pittsburgh Medical Center, Pittsburgh, PA 15213, USA

**Keywords:** extramedullary spinal cord tumors, intraoperative neurophysiological monitoring, IONM, evoked potentials

## Abstract

**Background/Objectives:** Intraoperative neurophysiological monitoring (IONM) is used to detect and prevent neurological injury during extramedullary spinal cord tumor (EMSCT) resection, but its diagnostic accuracy lacks systematic validation with recent evidence. This meta-analysis evaluates the performance of somatosensory evoked potentials (SSEPs), transcranial motor evoked potentials (TcMEPs), and multimodal (SSEP + TcMEP) IONM in predicting deficits during EMSCT resections. **Methods:** Following PRISMA-DTA guidelines, we searched MEDLINE, PubMed, and Ovid (inception to April 2025) for studies on IONM in EMSCT surgeries (PROSPERO: CRD420251047345). Pooled sensitivity, specificity, and reversibility metrics were calculated using bivariate models, with quality assessed via QUADAS-2. Z-test and Bayesian meta-analysis were used for comparisons. **Results:** Across 20 studies (2672 patients), multimodal IONM showed a log DOR of 4.310 (95% CI: 3.581–5.039) and an AUC of 94.2%, TcMEP monitoring showed a log DOR of 4.367 (95% CI: 3.765–5.127) and an AUC of 92%, while SSEP monitoring showed a log DOR of 3.463 (95% CI: 2.702–4.224) and an AUC of 82%. All modalities demonstrated high specificity (>95%), indicating low false-positive rates. Bayesian analysis revealed >90% probability that TcMEP-based approaches were superior to SSEPs. Reversible TcMEP changes were associated with an 11% (95% CI: 4–24%) postoperative deficit rate, compared to 35% (95% CI: 12–67%) for SSEPs. **Conclusions:** These findings caution against relying solely on SSEPs and support the use of multimodal IONM strategies, which enhance early detection of impending neurological injury, enable timely surgical interventions, and help prevent permanent neurological damage in EMSCT resections. Although TcMEP and multimodal monitoring showed similar diagnostic accuracy, we continue to recommend multimodal approaches as the current standard of care, pending prospective studies to determine if TcMEP alone can reliably replace multimodal monitoring.

## 1. Introduction

Spinal cord tumors represent a diverse group of pathologies with varying degrees of surgical complexity and risk [1]. Extramedullary spinal cord tumors (EMSCTs), account for approximately 40% of all spinal tumors, with an estimated annual incidence ranging from 0.4 to 1.1 per 100,000 population worldwide [2]. Intramedullary spinal cord tumors (IMSCTs), located within the cord parenchyma, are associated with the highest operative risk due to their proximity to major white matter tracts [3]. By contrast, EMSCTs generally have more favorable outcomes but still pose significant challenges, with new or worsened neurological deficits reported in about 4.9–6% of patients postoperatively, potentially due to displacement or compression of neural structures within a narrowed spinal canal [4,5,6].

Intraoperative neurophysiological monitoring (IONM) has become a cornerstone in the surgical management of IMSCTs, where its value has been extensively studied, including in our prior work [7,8,9]. Despite their relatively higher incidence compared to IMSCTs, the diagnostic utility of IONM modalities in EMSCT surgery remains less clearly established. Somatosensory evoked potentials (SSEPs) assess sensory pathway integrity by recording cortical responses to peripheral nerve stimulation. Transcranial motor evoked potentials (TcMEPs) evaluate motor pathway function via transcranial stimulation of the motor cortex and muscle response recording. A multimodal approach combines both SSEPs and TcMEPs to provide a more comprehensive assessment of neural function during surgery [10,11]. Several studies have evaluated these techniques in EMSCTs; however, these studies are often limited by modest sample sizes, and the relatively low incidence of EMSCTs and lower postoperative deficit rates further limit the power of individual studies to precisely estimate diagnostic performance [1,4,10,12]. A previous meta-analysis by Ishida et al. in 2019 provided important early insights but was constrained by a limited number of eligible cohorts [10]. Since then, more prospective and retrospective data have become available.

This raises a critical clinical question: How accurately do different IONM modalities, SSEP, TcMEP, and multimodal strategies, predict postoperative neurological deficits in EMSCT surgeries?

In this context, we conducted a systematic review and meta-analysis to evaluate and compare the diagnostic performance of these modalities. We also explored the predictive value of reversibility in IONM changes. By consolidating current evidence, this study aims to clarify the diagnostic utility of IONM in EMSCT resections and support more informed intraoperative decision-making.

## 2. Materials & Methodology

### 2.1. Protocol and Registration

This systematic review was conducted in accordance with the Preferred Reporting Items for Systematic Reviews and Meta-Analyses of Diagnostic Test Accuracy Studies (PRISMA-DTA) guidelines [13] (PRISMA checklist is provided in Supplementary Files). The protocol was registered on the International Prospective Register of Systematic Reviews (PROSPERO) on 1 April 2025, prior to the initiation of data extraction, under the registration number [CRD420251047345]. The review was conducted in strict adherence to the registered protocol, with no deviations made during the course of the study.

### 2.2. Literature Search

A comprehensive literature search was conducted across the following electronic databases: MEDLINE, PubMed, and Ovid (including the Cochrane Library and Embase), from their inception to 30 April 2025. The search was limited to articles published in English. To ensure a broad and inclusive search, we used a combination of keywords and Medical Subject Headings (MeSH) related to intraoperative neurophysiological monitoring (IONM) and spinal cord tumors. The full search strategy for PubMed is provided in Supplementary File S1. Importantly, the search was intentionally broad and not restricted specifically to extramedullary spinal cord tumors. Later, EMSCTs were identified manually. Additionally, we manually screened the reference lists of included articles to identify any potentially eligible studies not captured by the database search.

### 2.3. Study Selection

**Figure 1:**
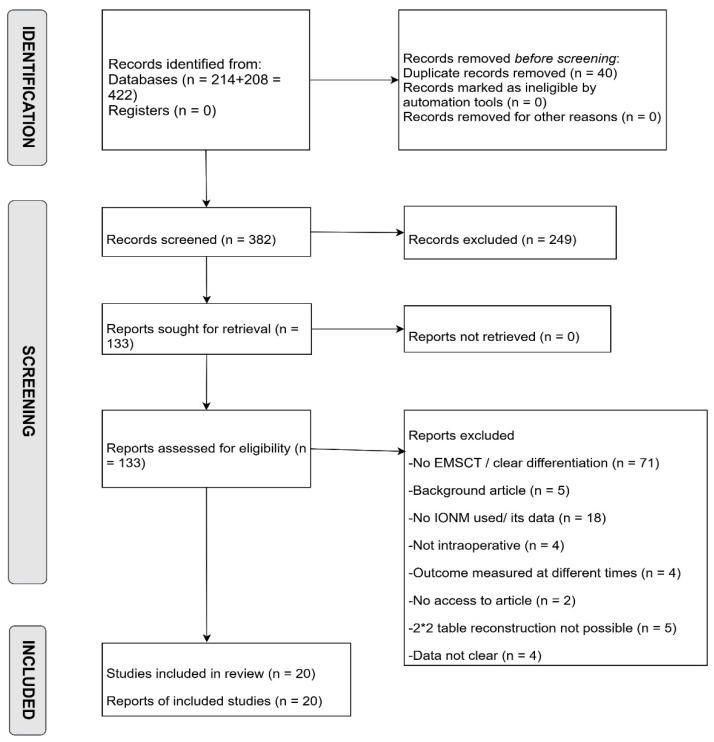
PRISMA Flow diagram summarizing the study selection.

All retrieved records were uploaded to Rayyan, where duplicates were removed. Initial title and abstract screening was performed independently and blindly by two authors to exclude clearly irrelevant studies, reviews, and case reports. Eligible articles then underwent full-text review based on predefined eligibility criteria, with exclusion reasons documented. In the final stage, during data extraction, additional studies were excluded due to unclear or incomplete reporting of IONM-related outcomes. After unblinding, conflicts at any stage were resolved through discussion and guidance. The complete study selection process is presented in the PRISMA flow diagram (Figure 1).

### 2.4. Inclusion and Exclusion Criteria

We included prospective, retrospective studies, observational cohorts, and randomized controlled trials that evaluated the role of IONM during surgical resection of EMSCTs. To ensure sufficient data quality and sample size, studies were required to report on at least 10 EMSCT resections. This decision was made because studies with smaller sample sizes, such as case series or case reports, often presented unique or atypical findings and are prone to bias.

Eligible studies had to utilize TcMEPs, SSEPs, EMG, or any combination of these modalities (i.e., multimodal IONM). Furthermore, studies were required to report immediate postoperative neurological status (prior to discharge) compared to preoperative assessments, using standardized clinical assessments such as the Modified McCormick Scale or comparable functional grading tools.

Studies were excluded if they did not correlate intraoperative IONM changes with postoperative neurological outcomes, involved non-human subjects, were published in languages other than English, or were case reports or review articles.

### 2.5. Data Extraction

Data extraction was performed independently by two authors, with any disagreements resolved through discussion and guidance. The following data were extracted from each study: author, year of publication, IONM modalities used (e.g., TcMEPs, SSEPs, EMG), threshold criteria for alarm values, and the correlation of IONM changes with postoperative neurological deficits. Deficits were classified into true positives (TP), false positives (FP), true negatives (TN), and false negatives (FN).

- TP: IONM changes that persisted despite intraoperative interventions and resulted in a postoperative neurological deficit.
- FP: IONM changes that persisted despite intraoperative interventions and did not result in a postoperative neurological deficit.
- TN: No IONM changes or no postoperative deficit.
- FN: No IONM change, but a postoperative deficit occurred.

For the purposes of this review, outcomes were defined as new postoperative neurological deficits, based on the McCormick scale or similar functional grading systems, as reported in individual studies. A multimodal test was considered positive if there was an irreversible change in any of the IONM modalities used. Reversible IONM changes, defined as amplitude reductions exceeding the study-specific thresholds that subsequently returned to baseline during surgery, were excluded from the analysis if the study treated them as rescue cases; otherwise, classified as test negatives.

### 2.6. Quality Assessment

The quality of the included studies was assessed using the QUADAS-2 tool, which evaluates the risk of bias and applicability of diagnostic accuracy studies. One author independently conducted the quality assessments, and the results were then verified and signed off by a second author to ensure accuracy and consistency. The details of each study’s quality assessment are provided in the Supplementary File S2.

### 2.7. Statistical Analysis

All statistical analyses were performed using R Studio (version 4.4.2). Pooled sensitivity, specificity, and area under the curve (AUC) for each IONM modality and the multimodal approach were calculated using the Reitsma random-effects and bivariate model, implemented via the mada package. Pooled log diagnostic odds ratios (log DOR) were estimated using the rma function from the metafor package, employing a random-effects model. Heterogeneity was assessed using the I^2^ statistic. Positive and negative likelihood ratios (PLR and NLR) were pooled using the metagen function from the meta package. Sensitivity analyses were conducted using the metaprop function, and the metainf function was used to assess the influence of individual studies by sequentially omitting each one. Fagan’s nomogram was applied to determine pre- and post-test probabilities using the Nomogrammer function from the petersenlab package. A subgroup analysis was conducted based on the amplitude threshold criteria used for TcMEP alerts to explore the effect of different alarm definitions on diagnostic performance and heterogeneity. The reversibility metrics were pooled across studies using a random-effects model.

Publication bias was assessed via funnel plots for visual inspection of asymmetry and Egger’s test for statistical confirmation of bias. When evidence of publication bias was detected, a trim-and-fill analysis was conducted using the trimfill function in the metafor package to adjust for potentially missing studies. To compare the diagnostic accuracy of different modalities, the Z-test was employed, with p-values reported for statistical significance. Finally, Bayesian meta-analysis was performed using the Bayesmeta package to estimate the probability that one test performs better than the other.

## 3. Results

### 3.1. Study Selection

The database search retrieved 422 records. After removing 40 duplicates, 382 unique articles were screened by title and abstract, leading to 133 full-text articles assessed for eligibility. Of these, 20 met the inclusion criteria and are included in the final analysis.

Not all included studies contributed data to every IONM modality; each study was incorporated into the relevant analysis based on the specific monitoring techniques evaluated. The study selection process is detailed in the PRISMA flow diagram (Figure 1).

### 3.2. Study Characteristics

**Figure 2:**
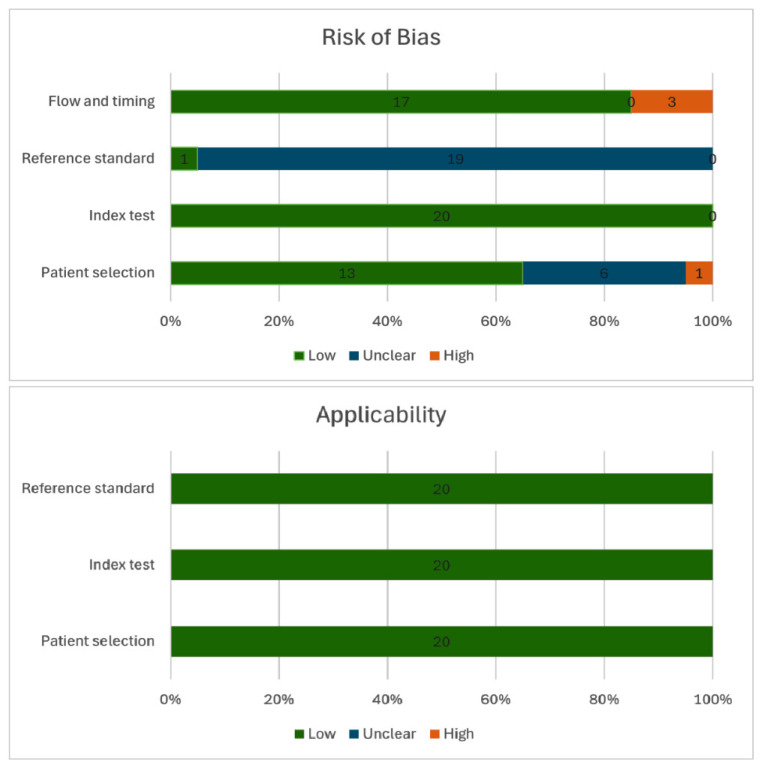
Bar chart summarizing the QUADAS-2 assessments.

A total of 20 studies published between 2007 and 2024 were included in the final analysis, encompassing 2672 patients undergoing resection of EMSCTs. Of these, 14 studies focused specifically on intradural extramedullary tumors, while 6 included extramedullary tumors in general. Fourteen studies were retrospective in design, and six were prospective. Quadas-2 quality assessments of each study are in Supplementary File S2 and outlined in Figure 2.

The use of IONM varied across studies:

- SSEPs were used in 10 studies, which comprised 653 patients.
- TcMEPs were used in 16 studies, which comprised 2302 patients.
- Multimodal monitoring (combining two or more IONM modalities) was used in 9 studies, which comprised 731 patients.

Characteristics of each study are summarized in Table 1 and detailed in Supplementary File S3.

Note: The total number of cases and studies is greater than the sum of individual modalities because studies that report on multiple IONM modalities or use multimodal monitoring are counted once for each relevant modality.

### 3.3. SSEP Monitoring

Among 653 patients that were monitored with SSEPs, significant and irreversible intraoperative changes were observed in 59 patients (9.0%), of which 41 patients (69.5%) developed new postoperative neurological deficits. Additionally, 43 patients (6.6% of the total cohort) developed deficits despite no significant intraoperative SSEP changes. Detailed diagnostic metrics are presented in Table 2.

### 3.4. TcMEP Monitoring

Among 2302 patients that were monitored with TcMEPs, significant and irreversible intraoperative changes were observed in 228 patients (9.9%), of which 130 patients (57.0%) developed new postoperative neurological deficits. Additionally, 52 patients (2.3% of the total cohort) developed postoperative deficits despite no significant intraoperative TcMEP changes. Comprehensive diagnostic metrics are presented in Table 2.

To address moderate heterogeneity observed in TcMEP data, a subgroup analysis was performed based on the amplitude reduction thresholds used to define a positive alarm (50%, 60%, and 70%). This analysis, detailed in Supplementary File S4, revealed no significant differences in overall diagnostic performance across threshold groups.

### 3.5. Multimodal Monitoring

Among 731 patients that were monitored with multimodal IONM across 9 studies, significant and irreversible intraoperative changes in at least one monitoring modality were observed in 79 patients (10.8%), of which 61 patients (77.2%) developed new postoperative neurological deficits. Additionally, 23 patients (3.1% of the total cohort) developed postoperative deficits despite no significant changes in any modality. Detailed diagnostic metrics are presented in Table 2.

### 3.6. Comparisons

**Figure 3:**
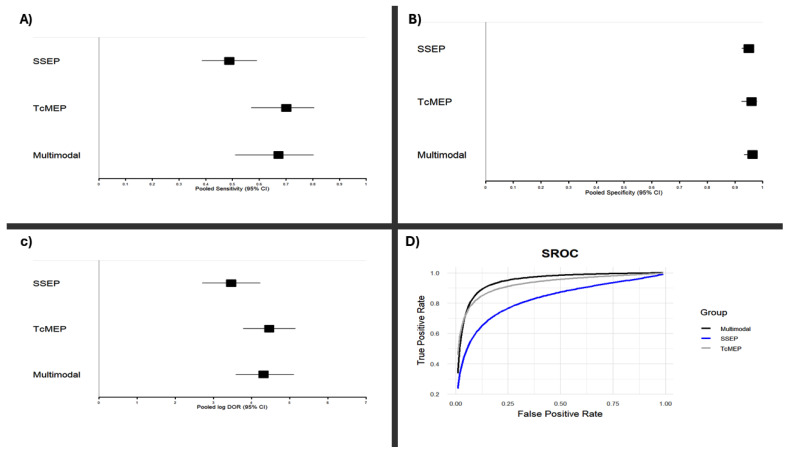
Pooled diagnostic performance estimates for intraoperative neurophysiological monitoring (IONM) modalities in EMSCT surgery. (**A**) Pooled sensitivity (95% CI); (**B**) Pooled specificity (95% CI); (**C**) Log diagnostic odds ratio (DOR) (95% CI); (**D**) Summary receiver operating characteristic (SROC) curves.

While traditional Z-tests applied to the pooled log diagnostic odds ratios (log DORs) did not reveal statistically significant differences in performance between monitoring modalities, estimates of the area under the curve (AUC) revealed a trend: Multimodal > TcMEP > SSEP (Figure 3).

**Figure 4:**
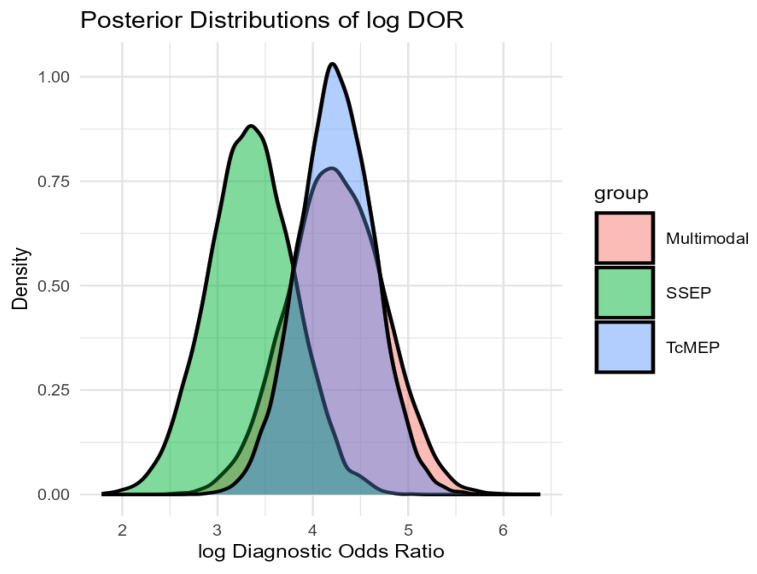
A visual representation of the log DOR estimates and their confidence intervals for each modality.

To further investigate potential differences, we performed a Bayesian meta-analysis, which showed that both TcMEP and multimodal intraoperative monitoring had over 90% posterior probability of superior diagnostic accuracy as compared to that of SSEP alone. TcMEP and multimodal monitoring exhibited largely overlapping posterior distributions, with similar mean log DORs and credible intervals, indicating comparable performance between them. These results underscore the relative strength of TcMEP-based strategies, while simultaneously highlighting the limitations of only SSEP monitoring during EMSCT resections. A visual representation of the log DOR estimates and their confidence intervals for each modality is presented in Figure 3 and Figure 4.

### 3.7. Reversibility of IONM Changes

Seven studies reported reversibility data for TcMEP monitoring [15,16,22,23,24,25,26]. The pooled incidence of reversible TcMEP changes was 46% (95% CI: 39–52%). Physiologically, reversible TcMEP changes during surgery indicate transient impairment of the motor pathways, likely due to reversible factors such as ischemia, mechanical traction, or anesthesia effects, that can recover if addressed promptly. Patients with reversible TcMEP changes had a pooled postoperative neurological deficit rate of only 11% (95% CI: 4–24%), suggesting that reversibility is generally associated with a favorable recovery prognosis.

Similarly, three studies reported reversibility data for SSEP monitoring [16,21,22]. The pooled incidence of reversible changes of 38% (95% CI: 16–67%). SSEP reversibility reflects transient sensory pathway disruption, often from similar reversible intraoperative insults. However, the postoperative neurological deficit rate among patients with reversible SSEP changes was higher 35% (95% CI: 12–67%), suggesting sensory pathway changes may have less predictable outcomes or reflect more severe or prolonged insults.

No publication bias was detected across any modality as confirmed by the symmetrical funnel plots and eggers test (See Supplementary File S5).

Panels (A) and (B) display forest plots of pooled sensitivities (A) and specificities (B) for somatosensory evoked potentials (SSEP), transcranial motor evoked potentials (TcMEP), and multimodal monitoring, derived from a random-effects meta-analysis of included studies. Panel (C) shows pooled log diagnostic odds ratios (Log DORs) for each modality using the same statistical method. Panel (D) displays the summary receiver operating characteristic (SROC) curves for each monitoring modality. These SROC curves were constructed using a bivariate random-effects model to jointly synthesize sensitivity and specificity estimates across studies. Results are based on extracted 2 × 2 contingency data from studies meeting the inclusion criteria as described in the Methods. Confidence intervals for all pooled values are indicated in each panel.

## 4. Discussion

The consistently high diagnostic odds ratios observed across all modalities confirm the valuable role of IONM during EMSCT resections to minimize postoperative neurologic deficits. When comparing individual modalities, TcMEPs and multimodal monitoring demonstrated comparable accuracy across all diagnostic metrics, and our Bayesian meta-analysis revealed a high posterior probability (>90%) suggesting that TcMEP-based monitoring approaches have better diagnostic odds ratio as compared to that of SSEP only monitoring. Specifically, TcMEP and multimodal strategies were more sensitive (70.1% and 67.2%, respectively) as compared to that of SSEP alone (48.8%) in detecting postoperative neurological deficits during EMSCT resections. This limited sensitivity of SSEP is in line with prior literature [1,15,16,20,28].

This superior performance of TcMEP-based approaches is best understood in light of spinal cord anatomy and the functional targets of each modality. TcMEPs monitor the descending motor pathways located in the anterior two-thirds of the spinal cord, whereas SSEPs assess the dorsal columns of the ascending sensory pathways located in the posterior third of the spinal cord [20]. Given the anatomical distribution of spinal cord damage, TcMEPs may be more sensitive to injury in broader regions, including the motor tracts, and could even detect damage to sensory pathways as well [30]. Moreover, TcMEPs are particularly sensitive to ischemia, changes in oxygenation, and blood pressure affecting the tracts, providing an early warning for surgeons to intervene [31,32]. The higher sensitivity of TcMEP is reflected in the reversibility data, with nearly 50% of TcMEP changes reversing with intervention, and only 11% of patients experiencing deficits post-intervention. In contrast, SSEP changes, although reversible in 40% of cases, still resulted in a higher postoperative deficit rate of 35%, suggesting less room for intraoperative intervention. This may be attributed to the slower acquisition of SSEP signals, which require averaging and thus delay the detection of acute intraoperative events like ischemia [14].

Interestingly, our analysis showed that TcMEP and multimodal monitoring had similar diagnostic accuracy. However, the apparent equivalence may not reflect true clinical interchangeability, as this statistical finding could be due to limited sample size or the lack of direct head-to-head comparisons between multimodal and TcMEP monitoring in homogeneous populations. Prospective studies directly comparing TcMEP and multimodal monitoring in similar patient cohorts are needed. While data on TcMEP versus multimodal monitoring equivalence is premature, the available evidence firmly supports avoiding SSEP-only monitoring [14,16,33,34].

Another notable finding is the consistently high specificity across all modalities, with SSEPs, TcMEPs, and multimodal monitoring all showed specificity values above 95%, indicating that when no significant IONM changes occur, the likelihood of a postoperative deficit is very low. Such high specificity can reassure surgeons during complex tumor dissections, especially when maneuvering near eloquent tracts. In practical terms, this enhances surgical confidence and may facilitate more complete resections without increasing the risk of neurological injury.

## 5. Conclusions

This meta-analysis reinforces the critical role of IONM during EMSCT resections. Among the evaluated modalities, TcMEP and multimodal monitoring demonstrated superior diagnostic accuracy over SSEP alone, particularly in sensitivity and early detection of neurologic injury. Although our results indicate similar diagnostic accuracy for TcMEP and multimodal monitoring, the absence of prospective, head-to-head comparisons limits definitive conclusions. Consequently, multimodal monitoring should continue to be the standard of care, while prospective trials are warranted to determine whether TcMEP alone could suffice or if multimodal strategies provide additional benefit. This presents an important area for future investigation.

## 6. Limitations

This study has several limitations. First, the population across different monitoring modalities was not homogeneous. The severity of postoperative deficits, tumor location within the spinal cord, and the distinction between transient and persistent deficits could not be analyzed due to insufficient detail in the source data. Furthermore, our inclusion criteria required studies to report on at least 10 EMSCT resections, which may have introduced a selection bias by excluding smaller case series. Only one study reported blinded postoperative neurological assessments, leaving the risk of bias for the reference standard unclear in most cases. Some patients may have been counted more than once across modality-specific analyses in mixed-modality studies, which could introduce overlap. Finally, the analysis of reversibility was limited by the small number of studies reporting relevant data, particularly for SSEP. The observed equivalence in diagnostic accuracy between TcMEP and multimodal monitoring should also be interpreted with caution, as it could be a result of limited sample sizes and a lack of direct head-to-head comparisons in the primary studies. Finally, restricting the review to English-language publications may also have led to the omission of relevant studies.

## 7. Future Research Recommendation

Future prospective studies should directly compare SSEP, TcMEP, and multimodal IONM in uniform EMSCT populations with adequate sample sizes. These studies should standardize definitions of intraoperative changes, report blinded and detailed neurological assessments and distinguish between transient and persistent deficits while also documenting severity and anatomical localization. The establishment of prospective registries and standardized outcome reporting templates would greatly enhance the quality and comparability of IONM research. Clinically, such high-quality data will guide real-time intraoperative decisions, enabling surgeons to better interpret neurophysiological signals, minimize irreversible injury, and maximize the safety and effectiveness of tumor resection.

## Figures and Tables

**Table 1:** Summary of characteristics of included studies.

	Sample Size	IONM Data Reported	Reference Standard
Vasileva et al., 2024 [4]	13	TcMEP	MRC grade
Mishra et al., 2024 [14]	32	TcMEP, SSEP, TcMEP + SSEP	Modified McCormick scale
Yu et al., 2023 [15]	87	TcMEP, SSEP, TcMEP + SSEP	Modified McCormick scale
Korn et al., 2015 [16]	97	TcMEP, SSEP, TcMEP + SSEP	Modified McCormick scale
Ghadirpour et al., 2019 [17]	108	TcMEP, SSEP, TcMEP + SSEP	Modified McCormick scale
Rho et al., 2016 [18]	160	TcMEP, SSEP	Modified McCormick scale
Kang et al., 2017 [19]	74	TcMEP, SSEP	MRC grade
Sutter et al., 2007 [20]	41	TcMEP, SSEP, TcMEP + SSEP	Motor deficits were given without reporting grading scale
Ghadirpour et al., 2015 [21]	68	TcMEP, SSEP, TcMEP + SSEP	Modified McCormick scale
Lakomkin et al., 2018 [1]	13	TcMEP, SSEP, TcMEP + SSEP	Deficits were reported without mentioning grading scale
Costa et al., 2013 [22]	59	TcMEP	Modified McCormick scale
Morito et al., 2023 [12]	29	TcMEP	Manual muscle Test
Yoshida et al., 2019 [23]	740	TcMEP	Motor deficits were given without reporting grading scale
Kobayashi et al., 2022 [24]	233	TcMEP	Manual muscle Test
Ushirozako et al., 2023 [25]	314	TcMEP	Manual muscle Test
Ando et al., 2020 [26]	244	TcMEP	Motor deficits were given without reporting grading scale
Siller et al., 2023 [27]	173	TcMEP + SSEP	McCormick scale
D’Ercole et al., 2023 [28]	55	SSEP	Modified McCormick scale
Ishida et al., 2019 [10]	103	TcMEP + SSEP	Modified McCormick scale
Harel et al., 2017 [29]	39	TcMEP + SSEP	McCormick scale

**Table 2:** Diagnostic accuracy of different IONM modalities.

	SSEP	TcMEP	Multimodal
Sensitivity (%) (95% CI)	48.8 (38.5–59.1)	70.1 (57.0–80.5)	67.2 (51.0–80.2)
Specificity (%) (95% CI)	95 (92.5–96.6)	96.0 (92.3–97.9)	96.3 (93.3–98.0)
Log DOR (95% CI)	3.463 (2.702–4.224)	4.367 (3.765–5.127)	4.310 (3.581–5.039)
DOR (95% CI)	31.93 (14.91–68.37)	83.14 (43.62–168.5)	79.77 (39.90–164.68)
AUC (%)	82	92	94.2
FPR (%) (95% CI)	5.0 (3.4–7.5)	4.0 (2.1–7.7)	3.7 (2.0–6.7)
FNR (%) (95% CI)	51.2 (40.9–61.5)	29.9 (19.5–43.0)	32.8 (19.8–49.0)
PLR (%) (95% CI)	13.91 (6.89–28.09)	15.16 (8.53–26.96)	15.26 (9.57–24.33)
NLR (%) (95% CI)	0.61 (0.44–0.85)	0.36 (0.25–0.52)	0.39 (0.25–0.63)
Heterogeneity (I^2^ %) (Zhou & Dendukuri, Holling’s, Holling’s sample size adjusted)	4.2, 0–0, 0–0.	8.3, 31.3–45, 3.4–4.1.	0, 16.8–29.6, 1.8–2.3.

## Data Availability

The original contributions presented in this study are included in the article and supplementary materials. Further inquiries can be directed to the corresponding author.

## Supplementary Materials

The following supporting information can be downloaded at: https://www.mdpi.com/article/10.3390/jpm15110513/s1, Supplementary File S1: Detailed search strategy, Supplementary File S2: Detailed QUADAS-2 assessments of included studies, Supplementary File S3: Detailed data extracted from each study, Supplementary File S4: Interpretations of meta-analysis and subgroup analysis, Supplementary File S5: Funnel plots across different monitoring modalities. Reference [35] is cited in the supplementary materials.
