# Supplementary material for: Precision in Practice: A Systematic Review and Meta-Analysis of Intraoperative Neurophysiological Monitoring for Optimizing Outcomes in Extramedullary Spinal Cord Tumor Resection
Source: J Pers Med. 2025 Oct 30;15(11):513. doi: 10.3390/jpm15110513 (PMC12653476; doi:10.3390/jpm15110513)
Supplement: Supplementary file 1 [file jpm-15-00513-s001.zip › supplementary file 1.pdf]

PUBMED:

((“Spinal Cord Neoplasms”[MeSH Terms] OR “Spinal cord tumor”[All Fields] OR “spinal cord neoplasm” [All Fields]) AND (“Intraoperative Neurophysiological Monitoring”[MeSH Terms] OR “monitoring, Intraoperative”[MeSH Terms] OR “neurophysiological monitoring”[All Fields] OR “IONM” [All Fields] OR “Evoked potentials, Motor”[MeSH Terms] OR “motor evoked potentials”[All Fields] OR “TcMEP”[All Fields] OR “MEPs”[All Fields] OR “muscle action potential”[All Fields] OR “D wave monitoring”[All Fields] OR “D wave”[All Fields] OR “Direct Wave”[All Fields] OR “D-Wave”[All Fields] OR “multimodal monitoring”[All Fields]))

Filters: English, Humans, Full text.

=214 papers

OVID:

1. Spinal cord tumors.mp. [mp=title, abstract, full text, caption text] = 1538
2. Spinal cord neoplasms.mp. [mp=title, abstract, full text, caption text] = 170
3. 1 or 2 = 1651
4. Intraoperative neurophysiological monitoring.mp. [mp=title, abstract, full text, caption text] = 794
5. D wave monitoring.mp. [mp=title, abstract, full text, caption text] = 47
6. Direct wave.mp. [mp=title, abstract, full text, caption text] = 328
7. d-wave.mp. [mp=title, abstract, full text, caption text] = 1357
8. motor evoked potentials.mo. [mp=title, abstract, full text, caption text] = 7648
9. MEP.mp. [mp=title, abstract, full text, caption text] = 10903
10. Neurophysiological monitoring.mp. [mp=title, abstract, full text, caption text] = 1680
11. TcMEP.mp. [mp=title, abstract, full text, caption text] = 207
12. 4 or 5 or 6 or 7 or 8 or 9 or 10 or 11 = 17406
13. 3 and 12 = 208

= **208 papers**

Final Total: 422 Papers
