# Supplementary material for: Precision in Practice: A Systematic Review and Meta-Analysis of Intraoperative Neurophysiological Monitoring for Optimizing Outcomes in Extramedullary Spinal Cord Tumor Resection
Source: J Pers Med. 2025 Oct 30;15(11):513. doi: 10.3390/jpm15110513 (PMC12653476; doi:10.3390/jpm15110513)
Supplement: Supplementary file 1 [file jpm-15-00513-s001.zip › Supplementary file 4.pdf]

TcMEP:

Bivariate diagnostic random-effects meta-analysis

Estimation method: REML

Fixed-effects coefficients

```
                  Estimate Std. Error      z Pr(>|z|) 95%ci.lb 95%ci.ub
tsens.(Intercept)    0.851      0.291  2.928    0.003    0.281    1.420  **
t□pr.(Intercept)    -3.170      0.353 -8.982    0.000   -3.861   -2.478 ***
sensitivity          0.701          -      -        -    0.570    0.805
□alse pos. rate      0.040          -      -        -    0.021    0.077
---
Signi□ codes:  0 '***' 0.001 '**' 0.01 '*' 0.05 '.' 0.1 ' ' 1
```

Variance components: between-studies Std. Dev and correlation matrix

```
      Std. Dev tsens  t□pr
tsens    0.823 1.000     .
t□pr     1.153 0.526 1.000
```

```
 logLik     AIC     BIC
 33.958 -57.915 -50.587
```

AUC: 0.92

Partial AUC (restricted to observed FPRs and normalized): 0.854

I2 estimates

Zhou and Dendukuri approach: 8.3 %

Holling sample size unadjusted approaches: 31.3 - 45 %

Holling sample size adjusted approaches: 3.4 - 4.1 %

Pooled LR+ = 15.16 (95% CI: 8.53 to 26.96)

Pooled LR- = 0.36 (95% CI: 0.25 to 0.52)

SSEP:

Bivariate diagnostic random-effects meta-analysis

Estimation method: REML

Fixed-effects coefficients

	Estimate	Std. Error	z	Pr(>|z|)	95%ci.lb	95%ci.ub	
tsens.(Intercept)	-0.049	0.213	-0.232	0.817	-0.468	0.369	
tfpr.(Intercept)	-2.937	0.215	-13.681	0.000	-3.357	-2.516	***
sensitivity	0.488	-	-	-	0.385	0.591	
false pos. rate	0.050	-	-	-	0.034	0.075	

Signif. codes: 0 ‘***’ 0.001 ‘**’ 0.01 ‘*’ 0.05 ‘.’ 0.1 ‘ ’ 1

Variance components: between-studies Std. Dev and correlation matrix

	Std. Dev	tsens	tfpr
tsens	0.000	1.000	.
tfpr	0.000	-0.627	1.000

logLik	AIC	BIC
28.677	-47.354	-42.376

AUC: 0.82

Partial AUC (restricted to observed FPRs and normalized): 0.429

I2 estimates

Zhou and Dendukuri approach: 4.2 %

Holling sample size unadjusted approaches: 0 - 0 %

Holling sample size adjusted approaches: 0 - 0 %

Pooled LR+ = 13.91 (95% CI: 6.89 to 28.09)

Pooled LR- = 0.61 (95% CI: 0.44 to 0.85)

Multimodal:

Bivariate diagnostic random-effects meta-analysis

Estimation method: REML

Fixed-effects coefficients

	Estimate	Std. Error	z	Pr(>|z|)	95%ci.lb	95%ci.ub	
tsens.(Intercept)	0.719	0.346	2.076	0.038	0.040	1.398	*
tfpr.(Intercept)	-3.263	0.323	-10.091	0.000	-3.897	-2.629	***
sensitivity	0.672	-	-	-	0.510	0.802	
false pos. rate	0.037	-	-	-	0.020	0.067	

Signif. codes: 0 ‘***’ 0.001 ‘**’ 0.01 ‘*’ 0.05 ‘.’ 0.1 ‘ ’ 1

Variance components: between-studies Std. Dev and correlation matrix

	Std. Dev	tsens	tfpr
tsens	0.639	1.000	.
tfpr	0.609	1.000	1.000

logLik AIC BIC

25.435 -40.870 -36.419

AUC: 0.942

Partial AUC (restricted to observed FPRs and normalized): 0.698

I2 estimates

Zhou and Dendukuri approach: 0 %

Holling sample size unadjusted approaches: 16.8 - 29.6 %

Holling sample size adjusted approaches: 1.8 - 2.3 %

Pooled LR+ = 15.26 (95% CI: 9.57 to 24.33)

Pooled LR- = 0.39 (95% CI: 0.25 to 0.63)

SUBGROUP ANALYSIS

<u>TcMEP 50%:</u>

Bivariate diagnostic random-effects meta-analysis

Estimation method: REML

Fixed-effects coefficients

	Estimate	Std. Error	z	Pr(>|z|)	95%ci.lb	95%ci.ub	
tsens.(Intercept)	0.766	0.303	2.532	0.011	0.173	1.360	*
t�pr.(Intercept)	-3.323	0.329	-10.095	0.000	-3.968	-2.678	***
sensitivity	0.683	-	-	-	0.543	0.796	
�alse pos. rate	0.035	-	-	-	0.019	0.064	

Signi� codes: 0 ‘***’ 0.001 ‘**’ 0.01 ‘*’ 0.05 ‘.’ 0.1 ‘ ’ 1

Variance components: between-studies Std. Dev and correlation matrix

	Std. Dev	tsens	t�pr
tsens	0.440	1.000	.
t�pr	0.566	1.000	1.000

logLik	AIC	BIC
26.415	-42.831	-38.379

AUC: 0.928

Partial AUC (restricted to observed FPRs and normalized): 0.719

I2 estimates

Zhou and Dendukuri approach: 0 %

Holling sample size unadjusted approaches: 0 - 0 %

Holling sample size adjusted approaches: 0 - 0 %

Pooled LR+ = 17.11 (95% CI: 9.95 to 29.42)

Pooled LR- = 0.38 (95% CI: 0.28 to 0.53)

<u>TcMEP 70:</u>

Bivariate diagnostic random-effects meta-analysis

Estimation method: REML

Fixed-effects coefficients

	Estimate	Std. Error	z	Pr(>|z|)	95%ci.lb	95%ci.ub

```
tsens.(Intercept)   1.376     0.743  1.851    0.064   -0.081    2.833   .
tfpr.(Intercept)   -2.629     0.770 -3.416    0.001   -4.138   -1.121 ***
sensitivity        0.798         -     -       -    0.480    0.944
false pos. rate     0.067         -     -       -    0.016    0.246
---
Signif. codes:  0 '***' 0.001 '**' 0.01 '*' 0.05 '.' 0.1 ' ' 1

Variance components: between-studies Std. Dev and correlation matrix
     Std. Dev tsens  tfpr
tsens    1.425 1.000     .
tfpr     1.657 0.272 1.000

logLik    AIC    BIC
 8.846 -7.693 -6.180

AUC:  0.935
Partial AUC (restricted to observed FPRs and normalized):  0.876

I2 estimates
Zhou and Dendukuri approach:  62.5 %
Holling sample size unadjusted approaches:  65.9 - 81.1 %
Holling sample size adjusted approaches:  8.6 - 12.8 %

Pooled LR+ = 11.49 (95% CI: 4.8 to 27.49 )
Pooled LR- = 0.23 (95% CI: 0.08 to 0.7 )
```

TcMEP 60:

Bivariate diagnostic random-effects meta-analysis

Estimation method: REML

```
Fixed-effects coefficients

                  Estimate Std. Error       z Pr(>|z|) 95%ci.lb 95%ci.ub
tsens.(Intercept)   -0.494      0.745 -0.663    0.507   -1.955    0.967
tfpr.(Intercept)    -3.864      1.015 -3.807    0.000   -5.853   -1.875 ***
sensitivity          0.379          -      -        -    0.124    0.724
false pos. rate      0.021          -      -        -    0.003    0.133
---
Signif codes:  0 '***' 0.001 '**' 0.01 '*' 0.05 '.' 0.1 ' ' 1

Variance components: between-studies Std. Dev and correlation matrix

      Std. Dev  tsens  tfpr
tsens    0.000  1.000     .
tfpr     0.000 -0.340 1.000

 logLik    AIC    BIC
  7.247 -4.493 -7.562

AUC:  0.728
Partial AUC (restricted to observed FPRs and normalized):  0.233

I2 estimates
Zhou and Dendukuri approach:  13.6 %
```

Holling sample size unadjusted approaches: 0 - 0 %

Holling sample size adjusted approaches: 0 - 0 %

Pooled LR+ = 454.1 (95% CI: 0.2 to 1025088)

Pooled LR- = 0.71 (95% CI: 0.42 to 1.2)

Bayesian metanalysis:

P(TcMEP > SSEP): 93 %

P(MULTI > SSEP): 90.6 %
