# Supplementary material for: Precision in Practice: A Systematic Review and Meta-Analysis of Intraoperative Neurophysiological Monitoring for Optimizing Outcomes in Extramedullary Spinal Cord Tumor Resection
Source: J Pers Med. 2025 Oct 30;15(11):513. doi: 10.3390/jpm15110513 (PMC12653476; doi:10.3390/jpm15110513)

Funnel plots across different IONM modalities:

1. **SSEP monitoring:**

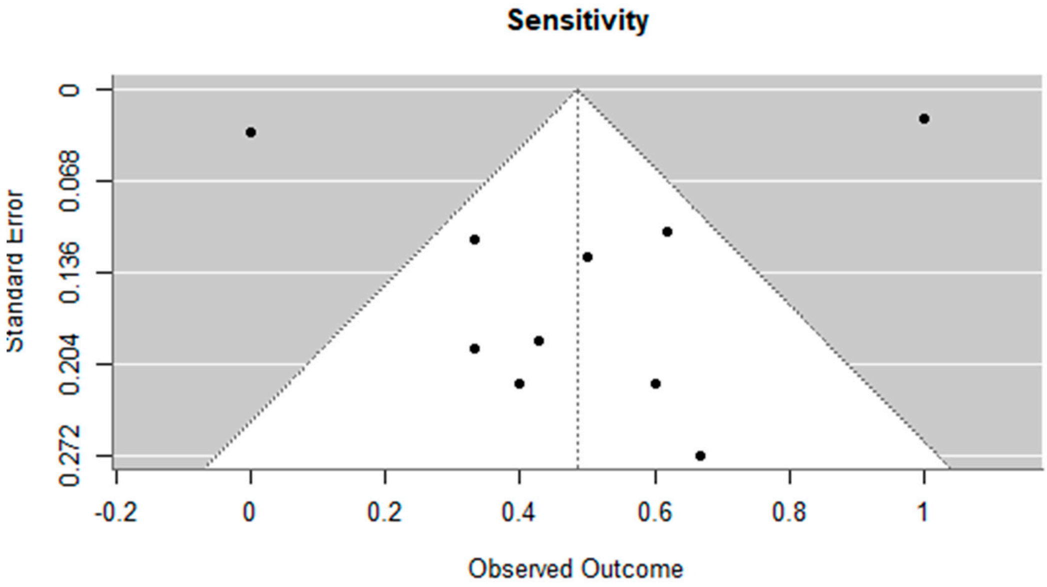

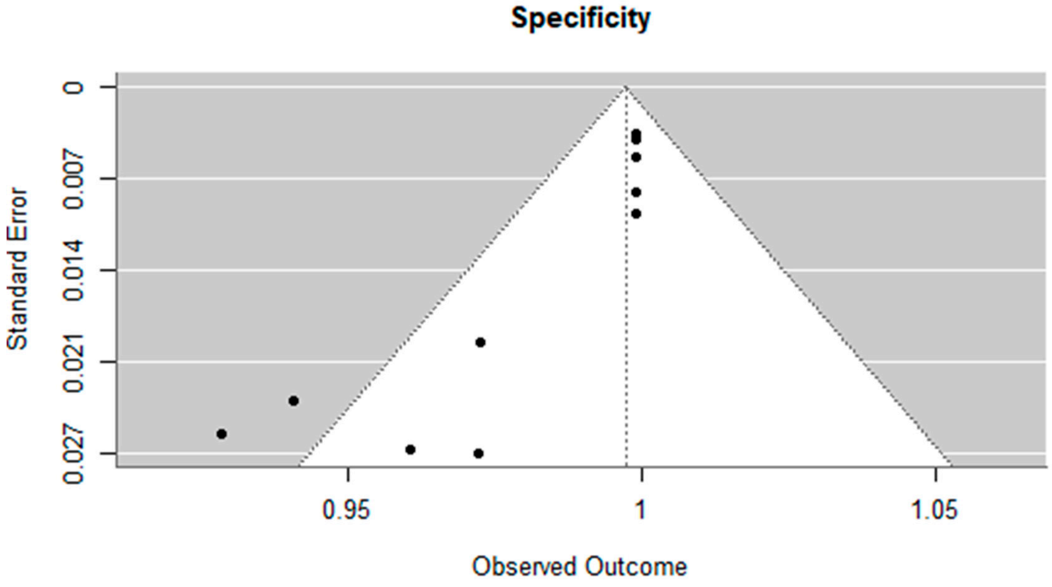

2. <u>TcMEP monitoring:</u>

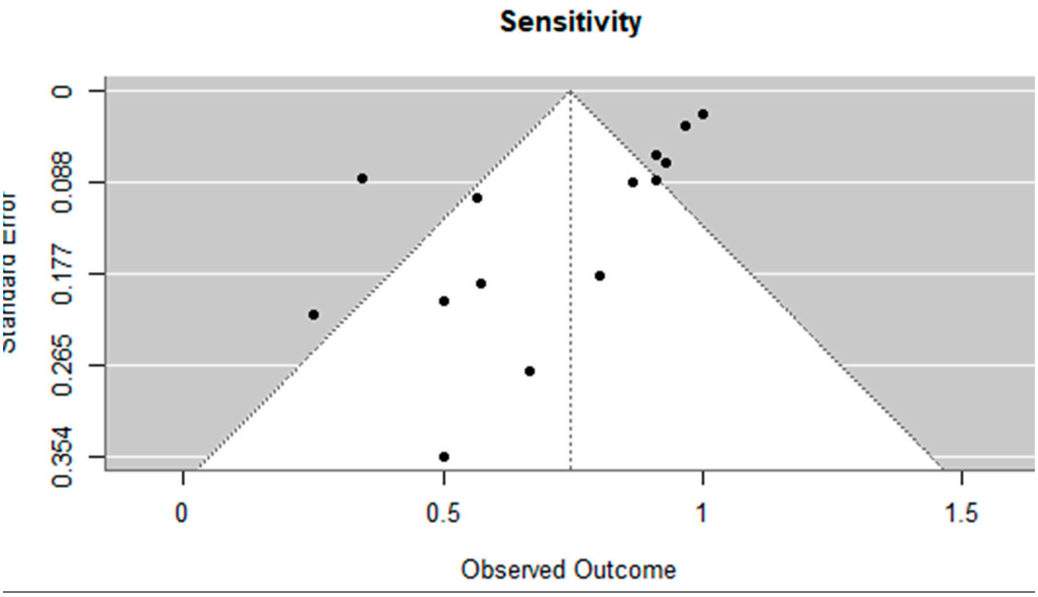

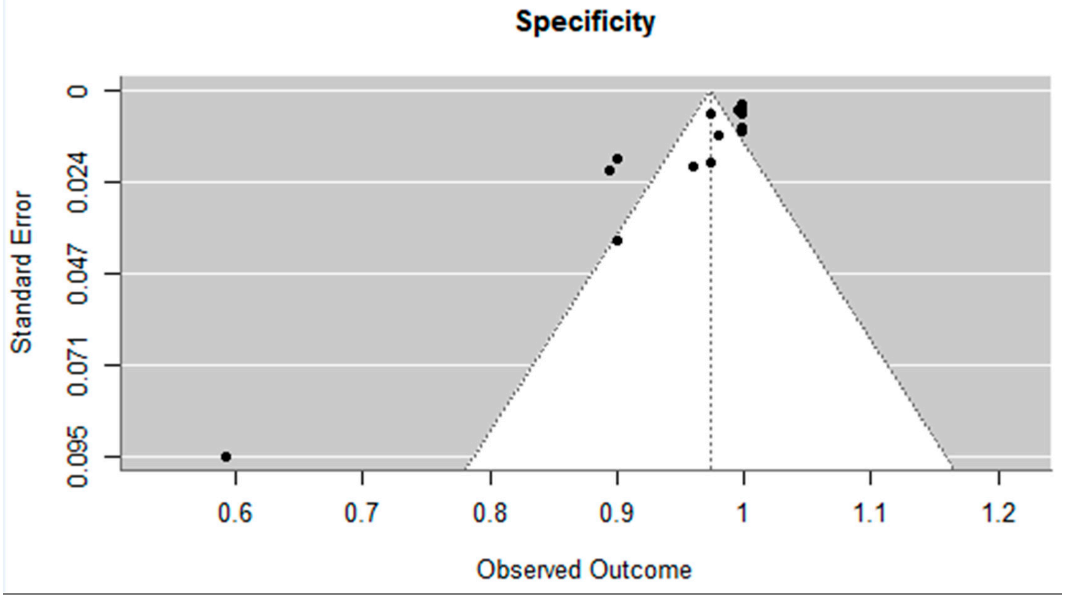

3. **<u>Multimodal IONM:</u>**

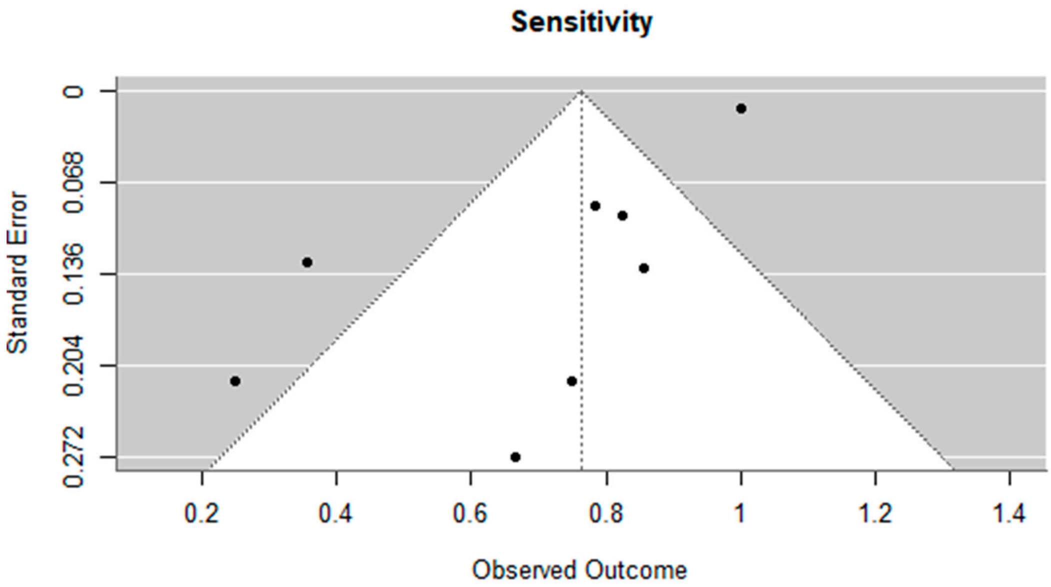

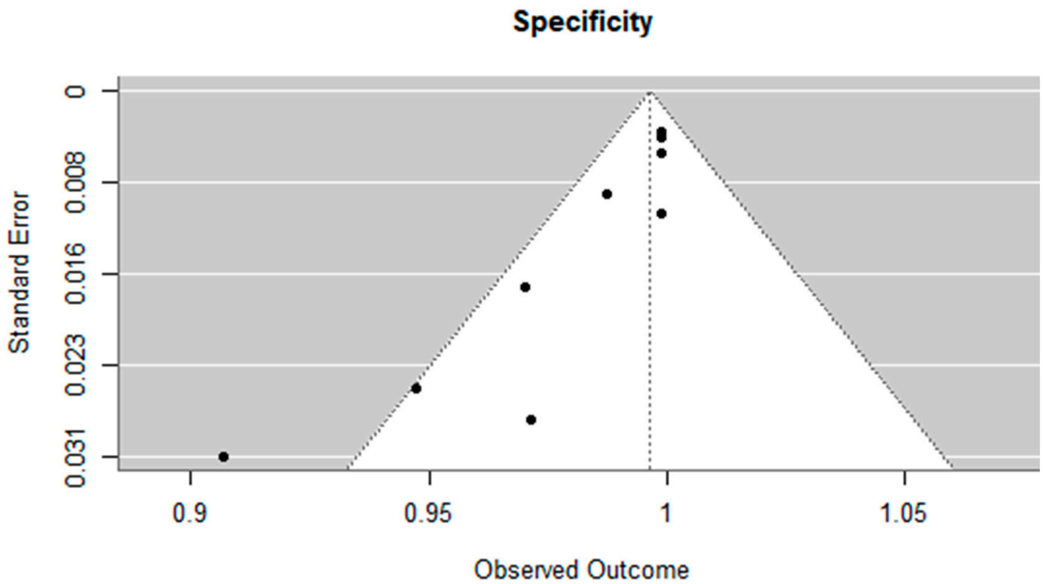

Supplement: Supplementary file 1 [file jpm-15-00513-s001.zip › supplementary file 5.pdf]
